# Reappraisal of Mental Illness-Related Beliefs During Psychiatric Clinical Exposure: Perceived Dangerousness, Ruminative Thought Style, and Cognitive–Metacognitive Characteristics

**DOI:** 10.3390/bs16081383

**Published:** 2026-08-12

**Authors:** İlker Güneysu, Seda Güneysu, Burcu Eser, Sare Aydın, Figen Ünal Demir, Mehmet Eren Yaşaran, Cansu Bak

**Affiliations:** 1 Department of Psychiatry, Faculty of Medicine, Tokat Gaziosmanpaşa University, 60250 Tokat, Turkey; 2 Department of Child and Adolescent Psychiatry, Faculty of Medicine, Tokat Gaziosmanpaşa University, 60250 Tokat, Turkey

**Keywords:** mental illness-related beliefs, perceived dangerousness, stigma, ruminative thought style, clinical exposure, cognitive–metacognitive characteristics

## Abstract

Stigma-related beliefs toward mental illness may change during psychiatric clinical exposure, but individual differences in this process remain insufficiently understood. This single-center observational pre–post study examined observed changes in mental illness-related beliefs among 185 medical interns who completed an approximately 30-day psychiatry clerkship between May 2025 and May 2026, with perceived dangerousness defined as the primary outcome. Beliefs were assessed before and after the exposure period using the Beliefs Toward Mental Illness Scale. Associations between baseline cognitive–metacognitive characteristics and observed change were evaluated using paired comparisons, linear mixed-effects models, and sensitivity analyses. Dangerousness scores decreased by the end of the exposure period, mean change = −2.66, 95% CI [−3.48, −1.81], q < 0.001, d_z_ = −0.47. Smaller decreases were observed in Helplessness and Deterioration in Interpersonal Relationships, whereas no significant change was found in the two-item Shame subscale. Higher baseline ruminative thought style was associated with a more limited decrease in dangerousness scores; B = 1.70, SE = 0.55, 95% CI [0.62, 2.77], q = 0.023. Although this association was consistent across sensitivity analyses, its incremental contribution was small (ΔR^2^_marginal_ = 0.017; f^2^ = 0.018 in the focused model). The finding should therefore be interpreted as a quantitatively modest association with observed belief change, not as a clinically meaningful predictor. Because the study included no concurrent control group or experimental manipulation and was conducted at one center, it does not establish a causal clerkship effect or support educational screening based on ruminative thought style.

## 1. Introduction

Stigma toward mental illness is not limited to general societal attitudes; it is a multidimensional process in which misinformation-based beliefs, prejudicial emotional responses, and discriminatory behaviors are closely intertwined. This process may adversely affect help-seeking behavior, access to healthcare, and participation in social life among individuals with mental illness (Clement et al., 2015; Corrigan & Watson, 2002; Knaak et al., 2017). Individuals with mental illness may face not only symptoms and functional impairment, but also processes of exclusion arising from established and often inaccurate beliefs about mental illness. For this reason, beliefs toward mental illness should not be examined only as a single global indicator of stigma, but also at the level of subdimensions that carry distinct cognitive and interpersonal meanings. Among these subdimensions, perceived dangerousness occupies a particularly central position because of its links with fear, avoidance, and social distance (Corrigan & Watson, 2002; Mannarini et al., 2022). These threat-related beliefs may also contribute to exclusionary responses that compound pre-existing psychosocial adversity through discrimination and restricted participation (Knaak et al., 2017; Thornicroft et al., 2016).

The perception of individuals with mental illness as dangerous, unpredictable, or difficult to control is one of the most salient components of public stigma (Corrigan & Watson, 2002). Such beliefs may be related, at the individual level, to threat appraisal and avoidance tendencies and, at the societal level, to increased social distance and the legitimization of discriminatory practices. In a large community sample examining stigma-related associations toward schizophrenia, Mannarini et al. (2022) reported that perceived dangerousness was associated with the desire for social distance and that social distance was in turn related to avoidance behavior. However, perceived dangerousness is not a fixed evaluation that emerges in the same way for all mental disorders or across all individuals. The diagnostic category, symptom patterns, social representations, previous contact experiences, and clinical observations may all contribute to the formation of this perception. Accordingly, perceived dangerousness represents a priority domain for understanding the psychological reappraisal of mental illness-related beliefs.

Psychiatric clinical exposure may provide a naturalistic context in which pre-existing beliefs about mental illness can be reconsidered. During the psychiatry clerkship, medical students and interns encounter not only theoretical knowledge but also clinical observation, patient interviews, ward rounds, and supervised assessments. These experiences may allow them to evaluate individuals with mental illness not only through diagnostic categories or social representations, but also in relation to personal histories, levels of functioning, treatment processes, and possibilities for recovery. Previous studies have reported decreases in some stigma-related attitudinal indicators following psychiatric education or clinical contact (Vilar Queirós et al., 2021; Zavorotnyy et al., 2023). Reductions in stigma indicators have also been shown to be more pronounced in educational practices that include patient-centered and reflective clinical experience (Hopp et al., 2023). Nevertheless, such findings do not indicate that clinical exposure produces change in the same direction or to the same extent in all students. How clinical experience is perceived, interpreted, and integrated with prior belief systems may differ across individuals.

The literature therefore provides a stronger basis for expecting average attitudinal improvement than for explaining heterogeneity in that improvement. The principal contribution of the present study is not another demonstration that stigma-related beliefs may decline during a psychiatry clerkship, but an examination of whether students exposed to the same routine curriculum differ in the extent of belief revision. This distinction shifts the theoretical question from who holds more stigmatizing beliefs to which characteristics may facilitate or constrain updating when students encounter information that is inconsistent with their prior assumptions.

Previous attempts to explain who changes during clinical exposure have been limited and have generally focused on demographic, contact, or educational characteristics rather than cognitive processing styles. In a controlled study of patient-centered clinical teaching involving psychiatric patients, Hopp et al. (2023) examined sex, previous contact with people with mental illness, interest in psychiatry or psychosomatics, and self-esteem. These factors were associated with baseline stigma, but none moderated stigma reduction. Zavorotnyy et al. (2023) reported that change in stigma covaried with willingness to pursue psychiatry, but did not test a baseline cognitive moderator of belief change. Longitudinal qualitative findings further suggest that the amount and quality of direct patient contact, students’ active role in consultations, supervisory support, and pre-existing beliefs may shape how perceptions evolve during a clerkship (Manley et al., 2024). Thus, heterogeneity in response has been recognized, but direct quantitative tests of baseline cognitive processing characteristics as moderators of within-person stigma-related change remain scarce.

Belief updating during clinical exposure requires attention to potentially disconfirming information, disengagement from established threat appraisals, and integration of patient-specific observations into broader representations of mental illness. Within this gap, ruminative thought style was selected as the focal cognitive candidate because it describes a content-independent tendency toward repetitive, persistent processing and difficulty disengaging from ongoing appraisals (Brinker & Dozois, 2009; Ehring & Watkins, 2008; Watkins, 2008). By comparison, psychological distress and negative automatic thoughts primarily index current affective or evaluative content, thought fusion concerns the significance and power attributed to thoughts, and the AnTI metacognition subscale captures worry-related metacognitive content (Hollon & Kendall, 1980; Lovibond & Lovibond, 1995; Wells, 1994; Wells et al., 2001). These constructs remain theoretically plausible and were examined in the same multivariable framework. The proposed distinction concerns conceptual proximity to the operation of belief updating; the study did not directly compare mechanisms or establish that rumination is globally more important than the alternatives.

This study examined observed change in beliefs toward mental illness during an approximately 30-day period of psychiatric clinical exposure among medical interns and evaluated whether that change was associated with baseline cognitive and metacognitive characteristics. Two hypotheses were specified. H1 was that dangerousness scores would decrease during the clinical exposure period. H2 was that higher baseline RTSQ scores would be associated with a more limited decrease in dangerousness scores. Other BTMI dimensions and interactions involving psychological distress, negative automatic thoughts, thought fusion, and worry-related metacognitive patterns were treated as complementary analyses. Because no concurrent control group was included, the hypotheses concerned observed longitudinal patterns and associated individual differences rather than a causal effect of the psychiatry clerkship.

## 2. Materials and Methods

### 2.1. Study Design and Context

This study was designed as a single-center, observational, longitudinal pre–post assessment to examine changes in beliefs toward mental illness during an approximately 30-day period of psychiatric clinical exposure among medical interns and to evaluate the association of these changes with baseline cognitive and metacognitive characteristics. The study was conducted with sixth-year medical students who completed their psychiatry clerkship at Tokat Gaziosmanpaşa University Faculty of Medicine between May 2025 and May 2026.

The psychiatry clerkship lasts approximately 30 days and combines theoretical instruction with clinical practice. During the clerkship, students receive training in core clinical skills, including psychiatric history-taking, mental status examination, participation in inpatient ward rounds, and evaluation of clinical follow-up processes. The program also covers psychiatric emergencies, mood disorders, psychotic disorders, anxiety- and trauma-related disorders, cognitive disorders, basic psychopharmacology, psychoeducation, and relevant legal processes. No additional educational or anti-stigma intervention was implemented beyond the routine clerkship program. The approximately 30-day duration was determined by the institution’s standard curriculum rather than selected by the research team; it should therefore be understood as the observed curricular window, not as an experimentally validated exposure dose or minimum duration for attitudinal change.

The study did not include a concurrent control group. Therefore, differences between pre-clerkship and post-clerkship measurements were interpreted not as the causal effect of the psychiatry clerkship, but as longitudinal patterns of change observed during the psychiatric clinical exposure period.

### 2.2. Participants

A total of 205 sixth-year medical students who completed the psychiatry clerkship during the study period were assessed for eligibility. Twenty students who did not participate in the initial assessment at the start of the clerkship were excluded from the analytic sample. Thus, the baseline analytic sample consisted of 185 medical interns.

The inclusion criteria for the analyses were completion of the psychiatry clerkship during the study period, participation in the baseline assessment at the beginning of the clerkship, and availability of the measurements required for the relevant analysis. For the analytic cohort, exclusion occurred only when a student did not participate in the baseline assessment; no additional clinical or academic-performance exclusion criteria were applied. Because some students had block-level missing data for the cognitive and metacognitive scales, the sample size varied across models. In the focused RTSQ analysis, 170 students and 339 repeated observations were evaluated. Students with missing data for a given analysis were excluded only from the relevant model and were retained in other analyses for which the necessary data were available.

### 2.3. Procedure

Data were collected using printed forms at two prespecified assessment points: at the beginning of the clerkship, before the routine clinical exposure period (day 0), and at the end of the approximately 30-day clerkship period (day 30). The same day-0/day-30 schedule was applied sequentially across routine clerkship blocks conducted between May 2025 and May 2026. Beliefs toward mental illness were measured at both assessment points. Baseline cognitive and metacognitive characteristics were entered into the predictor analyses using scores obtained at the initial assessment.

To match pre-clerkship and post-clerkship forms, each student was assigned a study-specific, randomly generated code. The research team provided the codes, and the identity–code linkage was known only to the researcher responsible for this procedure. Data collection on day 30 of the clerkship was conducted by a different researcher who was unaware of participant identities or baseline data. After the forms were matched using study-specific codes, the identity–code key was deleted, and all analyses were conducted on a de-identified dataset.

The forms were completed in an environment that allowed participants to respond independently, protected privacy, and provided standardized conditions. Participation in the study was voluntary. Students were informed that their decision to participate or not participate would not affect their psychiatry clerkship evaluations or academic grades. To reduce potential power asymmetry, faculty members responsible for evaluating students’ clerkship performance were separated from the researchers involved in data collection and analysis. No academic incentive was attached to participation.

### 2.4. Measures

#### 2.4.1. Sociodemographic Information Form

A two-item form prepared by the researchers was used to record participants’ age and sex. These variables were evaluated as demographic covariates in the main analyses.

#### 2.4.2. Beliefs Toward Mental Illness Scale

The Beliefs Toward Mental Illness Scale (BTMI; Turkish: Ruhsal Hastalıklara Yönelik İnançlar Ölçeği, RHYİÖ) is a 21-item self-report instrument that assesses general beliefs toward individuals with mental illness (Bilge & Çam, 2008; Hirai & Clum, 2000). Items are scored from 0 to 5, with higher scores indicating more negative beliefs. The Turkish form consists of three subscales: Helplessness and Deterioration in Interpersonal Relationships, dangerousness, and Shame (Bilge & Çam, 2008). In this study, the dangerousness subscale was defined as the primary outcome; the Helplessness and Deterioration in Interpersonal Relationships subscale as a prioritized secondary outcome; the two-item Shame subscale as an exploratory outcome; and the total score as a complementary outcome.

For the Helplessness and Deterioration in Interpersonal Relationships subscale, the internal consistency coefficients were α = 0.85 and ω = 0.86 at baseline and α = 0.84 and ω = 0.84 at the end of the clerkship. For the dangerousness subscale, the internal consistency coefficients were α = 0.77 and ω = 0.78 at baseline and α = 0.79 and ω = 0.80 at the end of the clerkship. For the total score, the internal consistency coefficients were α = 0.90 and ω = 0.90 at baseline and α = 0.89 and ω = 0.90 at the end of the clerkship. For the Shame subscale, the inter-item correlation and Spearman–Brown coefficients were r_ii_ = 0.33 and SB = 0.50 at baseline and r_ii_ = 0.37 and SB = 0.54 at the end of the clerkship. Because of the limited internal consistency observed for the two-item Shame subscale, findings related to this subscale were interpreted only at an exploratory and descriptive level. They were not included in the primary inferences.

#### 2.4.3. Ruminative Thought Style Questionnaire

The Ruminative Thought Style Questionnaire (RTSQ; Turkish: Ruminatif Düşünce Biçimi Ölçeği, RDBÖ) is a 20-item, seven-point Likert-type self-report instrument assessing individuals’ general tendency toward ruminative thinking (Brinker & Dozois, 2009; Karatepe et al., 2013). Higher total scores indicate a more pronounced ruminative thought style. In this study, the baseline RTSQ total score was treated as the main predictor. The internal consistency coefficients were α = 0.94 and ω = 0.94 at baseline and α = 0.96 and ω = 0.96 at the end of the clerkship.

#### 2.4.4. Depression Anxiety Stress Scales—21

The Depression Anxiety Stress Scales—21 (DASS-21) is a 21-item, four-point Likert-type scale assessing symptoms of depression, anxiety, and stress (Lovibond & Lovibond, 1995; Yılmaz et al., 2017). In this study, the raw total score was used without multiplying the subscale scores by two. Higher scores indicate more pronounced psychological distress. The internal consistency coefficients were α = 0.89 and ω = 0.89 at baseline and α = 0.91 and ω = 0.91 at the end of the clerkship.

#### 2.4.5. Automatic Thoughts Questionnaire (ATQ-30)

The Automatic Thoughts Questionnaire (ATQ-30; Turkish: Otomatik Düşünceler Ölçeği, ODÖ-30) is a 30-item, five-point Likert-type scale assessing the frequency of negative automatic thoughts associated with depression (Aydın & Aydın, 1990; Hollon & Kendall, 1980). Higher scores indicate more frequent negative automatic thoughts. The internal consistency coefficients were α = 0.96 and ω = 0.96 at baseline and α = 0.97 and ω = 0.97 at the end of the clerkship.

#### 2.4.6. Thought Fusion Instrument

The Thought Fusion Instrument (TFI; Turkish: Düşünce Kaynaşması Ölçeği, DKÖ) is a 14-item self-report instrument that assesses metacognitive beliefs about the meaning and power of thoughts (Güneysu et al., 2021; Wells et al., 2001). Higher scores indicate more pronounced thought fusion. The internal consistency coefficients were α = 0.87 and ω = 0.88 at baseline and α = 0.90 and ω = 0.91 at the end of the clerkship.

#### 2.4.7. Anxious Thoughts Inventory

The Anxious Thoughts Inventory (AnTI; Turkish: Kaygı Düşünceleri Envanteri, KDE) is a 22-item, four-point Likert-type self-report instrument assessing the content and process dimensions of worry (Kart et al., 2020; Wells, 1994). In this study, the AnTI metacognitive subscale was evaluated as a complementary and exploratory metacognitive variable. Higher scores indicate more pronounced metacognitive worry patterns. The internal consistency coefficients for the AnTI total scale items were α = 0.93 and ω = 0.93 at baseline and α = 0.92 and ω = 0.92 at the end of the clerkship.

### 2.5. Statistical Analysis

Statistical analyses were conducted using R 4.5.1 (R Core Team, 2025). The psych, lme4, lmerTest, car, sandwich, lmtest, lavaan, semTools, simr, and pwr packages were used for data preparation, scoring, and analyses. Linear mixed-effects models were fitted using the lme4 and lmerTest packages, whereas latent variable analyses were conducted using lavaan (Bates et al., 2015; Kuznetsova et al., 2017; Rosseel, 2012).

The analytic approach was based on subscale-level change in the BTMI. Because the BTMI total score combines conceptually distinct content domains, it was treated as a complementary outcome. The two-item Shame subscale, which showed a marked floor effect, was evaluated only at an exploratory and descriptive level. Cronbach’s alpha and McDonald’s omega coefficients were reported together to assess internal consistency.

Before scoring, item response ranges, missing observations, and the consistency of scale scores with scores recalculated from item responses were checked. Continuous variables were summarized using means and standard deviations, and categorical variables using counts and percentages. Change between pre-clerkship and post-clerkship BTMI scores was evaluated using paired-samples *t* tests, 95% bootstrap confidence intervals based on 5000 resamples, and Cohen’s d_z_ effect size. To examine sensitivity to distributional assumptions, the Wilcoxon signed-rank test was used as a supportive analysis. The Benjamini–Hochberg false-discovery rate correction was applied to subscale-level comparisons (Benjamini & Hochberg, 1995).

To evaluate the association of the baseline cognitive and metacognitive characteristics with the change in BTMI scores, linear mixed-effects models with participant-specific random intercepts were fitted. The time variable was coded as 0 for the pre-clerkship assessment and 1 for the post-clerkship assessment; continuous predictors were z-standardized. The predictors were entered in two theory-informed blocks for joint model comparison. The first block included DASS-21 and ATQ total scores, representing current affective distress and negative evaluative content. The second block included the TFI total score, RTSQ total score, and AnTI metacognitive subscale score, representing repetitive or metacognitive processing. This block structure was an analytic grouping and did not imply equal theoretical priority among the three second-block variables. The time × RTSQ interaction was the focal predictor-specific test corresponding to H2, whereas interactions involving the other constructs were complementary. The main effect of each predictor and its interaction with time were included in the model. Nested models differing in fixed effects were compared using maximum-likelihood estimation, whereas final coefficients were reported from restricted maximum-likelihood models. Degrees of freedom were calculated using the Satterthwaite approximation. The Benjamini–Hochberg correction was applied to the time × predictor tests conducted for the two prioritized BTMI subscales. Multicollinearity among predictors was examined using variance inflation factors, tolerance values, and condition indices.

After a time × RTSQ interaction was identified for the dangerousness subscale, simple time slopes were calculated at low (−1 SD), mean, and high (+1 SD) levels of RTSQ. The robustness of the main finding was examined using an age- and sex-adjusted mixed model, a paired complete-sample model, ANCOVA models adjusted for baseline dangerousness score, HC3-robust standard errors, inverse probability weighting, and participant-level leave-one-out influential-observation analyses. The incremental contribution of the time × RTSQ interaction was quantified using the change in marginal R^2^ and f^2^.

Block-level joint missingness was observed in the cognitive and metacognitive scales. Multiple imputation was not applied because there were insufficient auxiliary variables to support imputation and because the missingness mechanism could not be definitively classified based on observed data. The main analyses were conducted using a complete-case approach; findings were tested using an inverse probability-weighted sensitivity analysis based on observed age, sex, and baseline BTMI subscale scores.

Measurement-error sensitivity was evaluated through a secondary ordinal latent change model. Detailed invariance testing, residual specifications, and item-level robustness analyses are reported in Supplementary Table S6; the main text focuses on whether the latent analysis altered the substantive inference (Wu & Estabrook, 2016).

Because the study was based on a completed longitudinal dataset, post hoc observed power analyses were not conducted. Instead, minimum detectable effect sizes were calculated based on the achieved sample sizes. In the sensitivity analyses, the approximate minimum detectable coefficient for the time × baseline RTSQ interaction in the focused linear mixed model was B = 1.20. In supportive analyses, the minimum detectable effect size was calculated as Cohen’s d_z_ = 0.208 for the paired pre–post comparison and Cohen’s f^2^ = 0.048 for the ANCOVA model adjusted for baseline dangerousness score. All tests were two-sided, and the level of statistical significance was set at 0.05. These calculations characterize the smallest effects detectable with the achieved samples and were not interpreted as post hoc evidence that the study was adequately powered for every null association.

## 3. Results

### 3.1. Participant Characteristics and Analytic Sample

Of the 205 medical interns who completed the psychiatry clerkship during the study period, 20 were not included in the analytic sample because they did not participate in the baseline assessment at the beginning of the clerkship. Among the 185 participants with baseline data, the mean age was 24.26 years (SD = 1.22), and 51.4% of the sample were women. Baseline cognitive and metacognitive scale scores, together with the number of missing observations for these variables, are presented in Table 1. Because Ruminative Thought Style Questionnaire (RTSQ) data were available for 170 participants, the focused RTSQ mixed model was based on 339 repeated observations from 170 participants.

### 3.2. Observed Change During Psychiatric Clinical Exposure

Observed changes in the subscales of the Beliefs Toward Mental Illness Scale (BTMI) between the pre-clerkship and post-clerkship assessments are presented in Table 2. A significant decrease was observed in dangerousness scores, the primary outcome variable, by the end of the clerkship period (mean change = −2.66; 95% CI [−3.48, −1.81]; q < 0.001). The effect size for this change was small to moderate; d_z_ = −0.47. A more limited decrease was also found in the Helplessness and Deterioration in Interpersonal Relationships subscale; mean change = −1.59, 95% CI [−2.84, −0.33], q = 0.021, d_z_ = −0.18. In contrast, no significant difference was found between pre-clerkship and post-clerkship scores on the Shame subscale (q = 0.820). Because of its two-item structure and limited internal consistency, this subscale was evaluated only at a descriptive and exploratory level. The BTMI total score, examined as a complementary outcome, was also lower at the end of the clerkship; mean change = −4.22, 95% CI [−6.16, −2.29], *p* < 0.001, d_z_ = −0.31.

### 3.3. Associations of Baseline Cognitive and Metacognitive Characteristics with Change

The associations of baseline cognitive and metacognitive characteristics with change in BTMI scores were examined using multivariable linear mixed-effects models. The results of the time × predictor tests conducted for the two prioritized subscales are presented in Table 3. No significant time × predictor interaction was detected between change in the Helplessness and Deterioration in Interpersonal Relationships subscale and baseline DASS-21, ATQ-30, TFI, RTSQ, or AnTI metacognitive subscale scores.

For the dangerousness subscale, however, the time × RTSQ interaction was significant; B = 1.70, SE = 0.55, 95% CI [0.62, 2.77], *p* = 0.002, q = 0.023. The positive interaction coefficient indicates that students with a more pronounced baseline ruminative thought style showed a more limited decrease in dangerousness scores. The interactions between time and the other cognitive and metacognitive predictors examined for the dangerousness subscale were not significant.

The theoretical block comparisons were consistent with this pattern. For the Helplessness and Deterioration in Interpersonal Relationships subscale, the incremental contribution of the cognitive and metacognitive blocks to the model could not be demonstrated. For the dangerousness subscale, the first block, which included the DASS-21 and ATQ-30 interactions, remained at the threshold of significance after false-discovery rate correction (q = 0.054). In contrast, the second block, which included the TFI, RTSQ, and AnTI metacognitive subscale interactions, made a significant contribution to model fit; Δχ^2^ = 9.59, Δdf = 3, q = 0.045; see Supplementary Table S1.

### 3.4. Change in Dangerousness Scores According to Levels of Ruminative Thought Style

**Figure 1:**
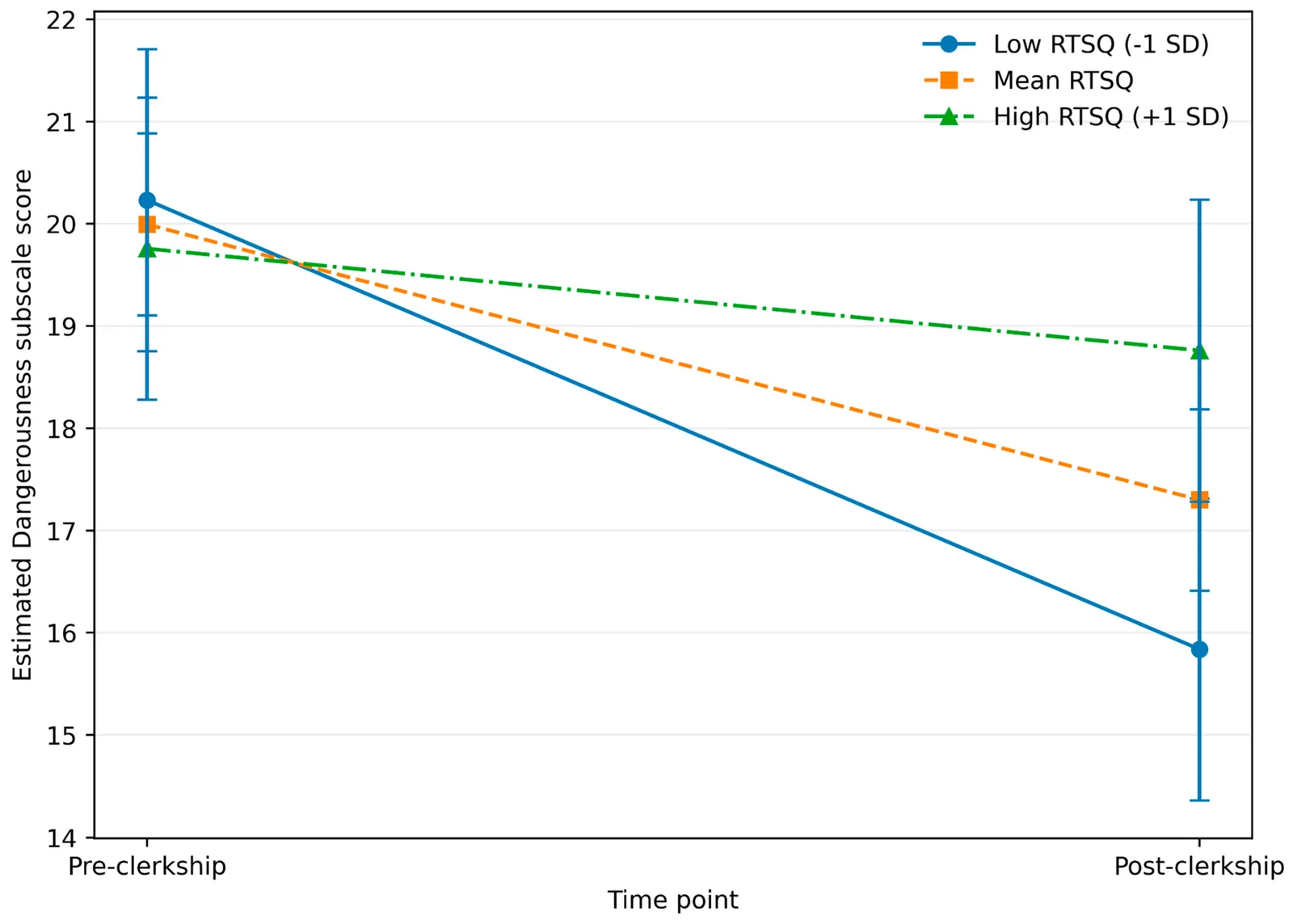
Estimated dangerousness subscale scores before and after psychiatric clinical exposure by baseline RTSQ level. Points and error bars represent marginal estimates and 95% confidence intervals derived from the focused linear mixed-effects model without age and sex adjustment; they are not raw group means. Dangerousness scores decreased more clearly at low and mean RTSQ levels, whereas the estimated decrease was smaller and not statistically significant at a high RTSQ level.

To improve the interpretability of the time × RTSQ interaction for the dangerousness subscale, simple time slopes were calculated at low, mean, and high baseline RTSQ levels. In the model adjusted for age and sex, the expected decrease in dangerousness scores was −4.38 points among students with low baseline RTSQ scores (−1 SD; 95% CI: [−5.76, −2.99]; *p* < 0.001). At the mean RTSQ level, the expected decrease was −2.77 points; 95% CI [−3.95, −1.59], *p* < 0.001. In contrast, among students with high baseline RTSQ scores (+1 SD), the expected decrease was more limited and not statistically significant: −1.16 points (95% CI [−2.68, 0.36]), *p* = 0.136. Marginal predictions derived from the primary mixed model visually supported the same pattern (Figure 1).

### 3.5. Focused Analyses and Sensitivity Checks

The time × RTSQ interaction for the dangerousness subscale remained generally consistent across alternative modeling approaches (Table 4). In the focused mixed model including all available observations, the interaction coefficient was B = 1.638, SE = 0.428, 95% CI [0.799, 2.476], *p* < 0.001. The findings did not materially change after adjustment for age and sex, restriction to participants with complete data at both time points, or use of ANCOVA models accounting for baseline dangerousness scores. In the extended ANCOVA model, baseline RTSQ score also retained its association with follow-up dangerousness score; B = 1.601, SE = 0.531, 95% CI [0.560, 2.642], *p* = 0.003.

Detailed predictor correlations, multicollinearity checks, missing-data comparisons, and the inverse probability-weighted analysis are reported in Supplementary Tables S2 and S3. None materially altered the RTSQ estimate, although selection related to unobserved characteristics remains possible.

Convergence, residual, and participant-level influence diagnostics are reported in Supplementary Table S4. The coefficient retained its direction after each leave-one-out deletion; however, the interaction added only ΔR^2^_marginal_ = 0.017 (f^2^ = 0.018), confirming that model stability did not translate into a large practical contribution.

### 3.6. Latent Variable Sensitivity Analysis

The secondary latent change analysis was directionally consistent with the observed-score result. Technical details of partial intercept invariance, category handling, residual covariances, and item-level robustness checks are provided in Supplementary Table S6 rather than repeated in the main text.

The time × RTSQ interaction for dangerousness was also supported in the more conservative Benjamini–Hochberg sensitivity correction in which the predefined analytic families were evaluated together; q = 0.011; see Supplementary Table S8.

## 4. Discussion

This study documented observed changes in mental illness-related beliefs during an approximately 30-day period of psychiatric clinical exposure among medical interns and identified an association between baseline ruminative thought style and change in dangerousness scores. The time × RTSQ coefficient retained its direction across alternative models and sensitivity analyses. The analytic stability indicates that the estimate was not dependent on a single modeling choice; it does not establish causality, replicability, predictive accuracy, or educational importance. The interaction accounted for only ΔR^2^_marginal_ = 0.017 (f^2^ = 0.018), and its practical contribution was therefore limited. In addition, without a concurrent control group, the average pre–post change and the moderator pattern may reflect repeated measurement, secular or cohort effects, experiences outside the clerkship, or unmeasured differences in how students encountered the curriculum. The result is best understood as a quantitatively modest association between a baseline processing style and observed within-clerkship change.

The decrease in perceived dangerousness is consistent with previous studies reporting more favorable attitudes after psychiatric education and clinical contact (Vilar Queirós et al., 2021; Zavorotnyy et al., 2023). It should therefore be interpreted mainly as a contextual replication of an established pattern rather than as the novel contribution of this study. Prior research suggests that direct patient interaction, supervised clinical experience, and reflective learning may accompany more favorable attitudinal change (Hopp et al., 2023). Longitudinal qualitative work likewise indicates that real-life patient contact, active participation in consultations, and supportive supervision can facilitate belief revision, whereas limited contact and durable pre-existing beliefs may constrain it (Manley et al., 2024). Brief or limited-contact approaches may produce less consistent effects (Papish et al., 2013). The present study extends this literature by examining the heterogeneity in change under routine clinical exposure, not by demonstrating that the clerkship itself caused the observed reduction.

The differentiation between subscales is important because beliefs toward mental illness do not constitute a uniform construct. Stigma-related beliefs may involve dangerousness, fear, avoidance, social distance, perceived treatability, and expectations regarding interpersonal functioning (Corrigan & Watson, 2002; Mannarini et al., 2022). Previous studies likewise indicate that attitudinal domains do not change uniformly after psychiatric education or contact-based experiences (da Rocha Neto et al., 2017; Kerby et al., 2008). Perceived dangerousness may be relatively amenable to reappraisal when students encounter patients, hear individual narratives, and observe treatment and recovery. By contrast, the Helplessness and Deterioration in Interpersonal Relationships dimension includes broader evaluations of illness course, functioning, and social participation, which may change less during a brief curricular period. The absence of significant change in Shame cannot establish resistance to change because this two-item subscale showed a floor effect and limited internal consistency.

Perceived dangerousness also has implications beyond individual attitudes. When threat-based stereotypes are translated into avoidance, restrictive practices, or unequal treatment, they may contribute to structural stigma, discrimination, and processes of oppression affecting people with mental illness (Knaak et al., 2017; Thornicroft et al., 2016; Zavorotnyy et al., 2023). The present study did not measure institutional practices, discrimination, or oppression directly; therefore, the observed reduction in dangerousness scores should not be interpreted as evidence that these broader processes changed during the clerkship.

The most distinctive finding was the association between baseline ruminative thought style and the magnitude of change in dangerousness scores. Rumination involves sustained engagement with ongoing appraisals and difficulty shifting away from established thought patterns (Ehring & Watkins, 2008; Mor et al., 2014; Watkins, 2008; Whitmer & Gotlib, 2012). In a clinical learning context, this processing style could make it harder to integrate patient-specific evidence that conflicts with prior threat-related assumptions. This mechanism was not measured directly, and the observed interaction does not show that rumination caused limited belief revision.

The comparative pattern across the measured constructs is consistent with, but does not prove, the proposed process distinction. Psychological distress and negative automatic thoughts may be more closely related to the emotional tone or content of baseline evaluations, whereas thought fusion and worry-related metacognition may affect the significance attributed to thoughts or the persistence of concern. Ruminative thought style is more directly aligned conceptually with sustained processing and disengagement, which provided the basis for its focal status in H2. This is an interpretive distinction rather than evidence that rumination is mechanistically superior. The nonsignificant interactions for the alternative constructs should not be interpreted as evidence that they are unimportant; limited power for small interaction effects, measurement overlap, and construct-specific reliability may have obscured associations. Replication with direct measures of cognitive flexibility, attentional shifting, and threat updating is required.

The small incremental effect materially constrains interpretation. Even if the association replicates, a variable explaining approximately 1.7% additional marginal variance would have limited standalone value for predicting which students will show attitudinal change. The study was not designed to establish an actionable threshold, classify students, or estimate individual educational benefit. Small effects can inform theory in educational research, but practical use requires replication and evidence that the association predicts durable behavioral outcomes (Kraft, 2020). Here, assessment ended with the clerkship and relied on self-report; persistence, social-distance behavior, and subsequent clinical conduct were not evaluated. The finding is therefore best treated as a hypothesis-generating signal about one possible source of heterogeneity, not as a clinically meaningful predictor.

Given the observational design, the educational and clinical-training implications are limited to testable intervention hypotheses. Future controlled studies could compare routine clerkship with structured reflective components, such as brief elicitation of expectations before patient contact, guided comparison of those expectations with patient-specific evidence, debriefing after emotionally salient encounters, and supervisor prompts that distinguish recurrent impressions from case-specific observations. Such trials should assess whether these components improve durable attitudes, social distance, standardized-patient behavior, or other clinically relevant outcomes. The present data do not show that these strategies are effective, do not support screening students by RTSQ score, and do not justify assigning differentiated curricula on the basis of ruminative thought style.

The strengths of this study include its examination of beliefs toward mental illness not only through a total score, but also at the level of subdimensions with theoretically distinct meanings. In addition, evaluating the association between baseline cognitive and metacognitive characteristics and change over time in a routine clinical training environment strengthens the ecological validity of the study. The preservation of the main finding across different analytic approaches, including adjusted mixed models, ANCOVA models accounting for baseline scores, inverse probability weighting, participant-level leave-one-out checks, and a latent change-score model accounting for measurement error, supports that the result was not dependent on a single modeling choice.

Several limitations should be considered. Most importantly, the observational pre–post design and the absence of a concurrent control group preclude attributing the observed changes specifically to the psychiatry clerkship or to any particular component of the curriculum. Repeated measurement, time-related change, social desirability, and experiences outside the clerkship remain plausible alternative explanations. Recruitment also occurred across successive routine clerkship blocks, and unmeasured variation in patient contact, clinical case mix, or supervision may have contributed to differences between cohorts.

The generalizability of the findings is limited by the inclusion of sixth-year medical interns from a single Turkish medical school and by the institution-specific approximately 30-day curriculum. The findings may therefore not extend directly to other stages of medical education, training programs, or cultural and healthcare settings. In addition, assessments were conducted only at the beginning and end of the clerkship and relied on self-report measures; consequently, the persistence of the observed changes and their correspondence with clinical behavior remain unknown. Finally, the proposed relevance of ruminative thought style to belief updating remains a theoretical interpretation, because cognitive flexibility, attentional disengagement, and threat-related updating were not directly measured.

Future research would benefit from controlled, multicenter designs comparing different educational programs or forms of clinical contact, longer follow-up periods, and the inclusion of measures of clinical behavior, social distance tendencies, or performance-based indicators alongside self-report assessments. Given the adverse effects of stigma on help-seeking, access to healthcare, and care processes, future studies should examine not only changes in scale scores, but also the extent to which attitudinal change is reflected in behavioral and clinical outcomes (Clement et al., 2015; Knaak et al., 2017). In addition, examining components such as the quality of patient contact, supervised clinical practice, educator feedback, and reflective learning sessions separately may better clarify the experiential processes through which change is shaped. Whether the association between ruminative thought style and change in perceived dangerousness replicates across different samples and whether possible intermediary processes such as cognitive flexibility, attentional shifting, or threat interpretation play a role in this association should also be evaluated.

## 5. Conclusions

This single-center observational pre–post study found that perceived dangerousness decreased during an approximately 30-day period of psychiatric clinical exposure among medical interns and that higher baseline ruminative thought style was associated with a more limited decrease. The association was consistent across several analytic approaches but small in incremental magnitude. It should therefore be understood as an associated individual characteristic, not as a clinically meaningful predictor, screening marker, or basis for differentiated education. Neither the average change nor the interaction can be attributed causally to the clerkship. Controlled multicenter studies across training stages and curricula, with longer follow-up and behavioral outcomes, are required before educational or clinical-training implications can be established.

## Figures and Tables

**Table 1:** Participant characteristics and baseline cognitive/metacognitive predictor scores.

Variable	*n* or *n* (%)	Missing *n*	M (SD)	Range
Age, years	185	0	24.26 (1.22)	22–31
Male	90 (48.6)	0	—	—
Female	95 (51.4)	0	—	—
DASS-21 total	169	16	14.31 (9.22)	0–39
ATQ total	170	15	51.86 (19.88)	30–133
TFI total	170	15	269.76 (194.96)	0–830
RTSQ total	170	15	70.07 (24.60)	20–131
AnTI metacognition subscale	169	16	6.44 (4.92)	0–22

Note. Among 205 students who completed the psychiatry clerkship between May 2025 and May 2026, 20 were excluded because they did not participate in the day-0 baseline assessment; the analytic sample comprised *N* = 185. Male participants are the reference category for sex. Continuous variables are reported as mean (standard deviation); categorical variables are reported as *n* (%). DASS-21 = Depression Anxiety Stress Scales—21; ATQ = Automatic Thoughts Questionnaire; TFI = Thought Fusion Instrument; RTSQ = Ruminative Thought Style Questionnaire; AnTI = Anxious Thoughts Inventory.

**Table 2:** Internal consistency and observed change in Beliefs Toward Mental Illness dimensions.

BTMI Dimension	n	t_0_ M (SD)	t_30_ M (SD)	Change M (SD)	Bootstrap 95% CI	t	*p*	q	d_z_
Helplessness and deterioration in interpersonal relationships	184	23.25 (9.96)	21.66 (9.16)	−1.59 (8.72)	[−2.84, −0.33]	−2.48	0.014	0.021	−0.18
Dangerousness	184	19.95 (6.14)	17.29 (6.08)	−2.66 (5.70)	[−3.48, −1.81]	−6.33	<0.001	<0.001	−0.47
Shame	184	1.08 (1.89)	1.11 (1.63)	0.03 (1.94)	[−0.24, 0.31]	0.23	0.820	0.820	0.02
BTMI total score	184	44.29 (15.65)	40.07 (14.72)	−4.22 (13.43)	[−6.16, −2.29]	−4.26	<0.001	—	−0.31

Note. Change was calculated as t_30_ − t_0_; negative values indicate lower scores at follow-up. q denotes the Benjamini–Hochberg-adjusted *p* value. The BTMI total score belongs to a separate complementary analysis family. d_z_ denotes Cohen’s d_z_ for paired observations. For the two-item Shame subscale, the inter-item correlation (r_ii_) and Spearman–Brown (SB) coefficient are reported. Given its limited internal consistency, the Shame subscale was interpreted only descriptively and exploratorily. BTMI = Beliefs Toward Mental Illness Scale.

**Table 3:** Baseline predictors of change in Beliefs Toward Mental Illness dimensions in multivariable mixed-effects models.

Outcome	Time × Predictor	B	SE	95% CI	df	t	*p*	q
Helplessness and deterioration in interpersonal relationships	DASS-21	0.48	0.96	[−1.41, 2.36]	167	0.50	0.621	0.674
	ATQ	1.08	1.05	[−0.97, 3.13]	167	1.03	0.302	0.504
	TFI	0.60	0.72	[−0.81, 2.00]	167	0.83	0.406	0.580
	RTSQ	0.98	0.87	[−0.73, 2.68]	167	1.12	0.264	0.504
	AnTI metacognition	−1.21	1.07	[−3.32, 0.89]	167	−1.13	0.261	0.504
Dangerousness	DASS-21	−0.26	0.61	[−1.44, 0.93]	167	−0.42	0.674	0.674
	ATQ	0.81	0.66	[−0.49, 2.10]	167	1.22	0.223	0.504
	TFI	−0.54	0.45	[−1.42, 0.35]	167	−1.19	0.235	0.504
	RTSQ	1.70	0.55	[0.62, 2.77]	167	3.09	0.002	0.023
	AnTI metacognition	−0.41	0.68	[−1.74, 0.92]	167	−0.61	0.544	0.674
BTMI total score	DASS-21	0.54	1.45	[−2.31, 3.39]	167	0.37	0.712	—
	ATQ	1.76	1.58	[−1.34, 4.86]	167	1.11	0.268	—
	TFI	−0.02	1.08	[−2.15, 2.10]	167	−0.02	0.982	—
	RTSQ	2.44	1.32	[−0.14, 5.02]	167	1.86	0.065	—
	AnTI metacognition	−1.44	1.63	[−4.63, 1.75]	167	−0.89	0.377	—

Note. Models are based on 167 participants and 334 repeated observations. B denotes the difference in t_0_-to-t_30_ score change per 1-SD increase in the corresponding baseline predictor. The 95% CIs are Wald confidence intervals. q values were calculated across the 10 interaction tests for the two prioritized subscales; the total score is complementary. BTMI = Beliefs Toward Mental Illness Scale.

**Table 4:** Focused RTSQ analyses and sensitivity models for dangerousness.

Analysis	*n*	Effect	Scale	Estimate	SE	95% CI	*p*
Observed-score dangerousness model, all available observations	170	Time × RTSQ	B	1.638	0.428	[0.799, 2.476]	<0.001
Age- and sex-adjusted observed-score model	170	Time × RTSQ	B	1.608	0.434	[0.758, 2.458]	<0.001
Observed-score model, paired complete sample	169	Time × RTSQ	B	1.568	0.429	[0.728, 2.408]	<0.001
Adjusted paired complete observed-score model	169	Time × RTSQ	B	1.544	0.435	[0.692, 2.395]	<0.001
ANCOVA: baseline dangerousness + RTSQ	167	RTSQ	B	1.516	0.358	[0.814, 2.219]	<0.001
ANCOVA: +age and sex	167	RTSQ	B	1.530	0.375	[0.795, 2.266]	<0.001
ANCOVA: full cognitive–metacognitive model	167	RTSQ	B	1.601	0.531	[0.560, 2.642]	0.003
Latent change-score model	169	Δ Dangerousness ~ RTSQ	β	0.337	0.089	[0.164, 0.511]	<0.001
IPW-weighted observed-score model	170	Time × RTSQ	B	1.649	0.427	[0.813, 2.486]	<0.001
Observed-score sensitivity with harmonized i13	170	Time × RTSQ	B	1.641	0.427	[0.803, 2.478]	<0.001
Latent change-score model without i13	169	Δ Dangerousness ~ RTSQ	β	0.345	0.093	[0.164, 0.527]	<0.001

Note. For observed-score mixed models, B denotes the difference in t_0_-to-t_30_ change per 1-SD increase in RTSQ. For ANCOVA models, B denotes the difference in follow-up dangerousness per 1-SD increase in RTSQ after adjustment for baseline dangerousness; HC3-robust standard errors are reported. Standardized beta is reported for latent models. In the focused observed-score model, the incremental contribution of the time × RTSQ interaction was ΔR^2^_marginal_ = 0.017 and f^2^ = 0.018, indicating a small effect size.

## Data Availability

The data supporting the findings of this study are available from the corresponding author upon reasonable request. The data are not publicly available because of privacy and ethical restrictions.

## Supplementary Materials

The following supporting information can be downloaded at https://www.mdpi.com/article/10.3390/bs16081383/s1, Table S1: Theory-informed block contributions to mixed-effects models; Table S2: Predictor correlations and multicollinearity diagnostics; Table S3: Missing-data evaluation and IPW sensitivity analysis; Table S4: Mixed-model diagnostics and influence analyses; Table S5: HC3-robust ANCOVA sensitivity model; Table S6: Latent measurement diagnostics and i13 robustness analyses; Table S7: Conditional latent change-score model; Table S8: Overall Benjamini–Hochberg sensitivity analysis; Table S9: Incremental effect size and power sensitivity analyses; Figure S1: Expected change in dangerousness by baseline RTSQ level in the age- and sex-adjusted model; Figure S2: Residuals versus fitted values for the final mixed-effects model; Figure S3: Normal Q-Q plot of residuals from the final mixed-effects model; Figure S4: Simulation-based sensitivity analysis for the focused mixed-effects model.
