# Supplementary material for: Reappraisal of Mental Illness-Related Beliefs During Psychiatric Clinical Exposure: Perceived Dangerousness, Ruminative Thought Style, and Cognitive–Metacognitive Characteristics
Source: Behav Sci (Basel). 2026 Aug 12;16(8):1383. doi: 10.3390/bs16081383 (PMC13509395; doi:10.3390/bs16081383)

Supplementary Tables and Figures

Supplementary Table S1

Theory-informed block contributions to mixed-effects models

Outcome	Model comparison	$\Delta\chi^2$	Δdf	p	q	AIC	BIC
Helplessness and deterioration in interpersonal relationships	Block 1: time × DASS-21 + time × ATQ	3.36	2	.186	.186	2380.16	2422.08
Helplessness and deterioration in interpersonal relationships	Block 2: time × TFI + time × RTSQ + time × AnTI metacognition	2.93	3	.403	.403	2383.23	2436.59
Dangerousness	Block 1: time × DASS-21 + time × ATQ	7.23	2	.027	.054	2080.90	2122.83
Dangerousness	Block 2: time × TFI + time × RTSQ + time × AnTI metacognition	9.59	3	.022	.045	2077.31	2130.67
BTMI total score	Block 1: time × DASS-21 + time × ATQ	5.89	2	.053	—	2671.95	2713.87
BTMI total score	Block 2: time × TFI + time × RTSQ + time × AnTI metacognition	3.71	3	.295	—	2674.24	2727.60

Note. Block comparisons were obtained from nested models estimated using ML. AIC and BIC values correspond to the model after the relevant block was added. q values reflect Benjamini-Hochberg correction across the four theory-informed block comparisons for the two prioritized subscales; the total score is complementary.

Supplementary Table S2

Predictor correlations and multicollinearity diagnostics

Panel A. *Pearson correlations among baseline cognitive and metacognitive predictors*

Variable	DASS-21	ATQ	TFI	RTSQ	AnTI metacognition
DASS-21	—	—	—	—	—
ATQ	0.66	—	—	—	—
TFI	0.28	0.14	—	—	—
RTSQ	0.56	0.55	0.32	—	—
AnTI metacognition	0.66	0.74	0.27	0.60	—

Note. The lower triangle is shown. Pairwise sample sizes range from 168 to 170.

Panel B. *Multicollinearity diagnostics*

Predictor	VIF	Tolerance
Baseline Dangerousness	1.17	0.86
DASS-21	2.22	0.45
ATQ	2.60	0.38
TFI	1.33	0.75
RTSQ	1.78	0.56
AnTI metacognition	2.86	0.35
Age	1.03	0.97
Female sex	1.14	0.88

Note. Maximum condition index = 3.57. Condition indices were calculated from the eigenvalue decomposition of the standardized predictor matrix. VIF, tolerance, and condition indices were evaluated jointly; no critical multicollinearity was identified.

Supplementary Table S3

Missing-data evaluation and IPW sensitivity analysis

Panel A. *Participants with and without available baseline RTSQ data*

Variable	RTSQ available: n; M (SD)	RTSQ missing: n; M (SD)	Hedges g	Welch p	q
Age	170; 24.28 (1.25)	15; 24.07 (0.96)	-0.18	.428	.951
DASS-21	169; 14.31 (9.22)	—	—	—	—
ATQ	170; 51.86 (19.88)	—	—	—	—
TFI	170; 269.76 (194.96)	—	—	—	—
AnTI metacognition	169; 6.44 (4.92)	—	—	—	—
BTMI total	170; 44.02 (15.46)	15; 45.27 (19.60)	0.08	.813	.951
Dangerousness	170; 19.88 (6.09)	15; 20.00 (7.41)	0.02	.951	.951
Helplessness and interpersonal deterioration	170; 23.05 (9.89)	15; 24.33 (11.74)	0.13	.687	.951
Shame	170; 1.09 (1.85)	15; 0.93 (2.34)	-0.08	.806	.951

Note. Sex distribution: 85 male and 85 female participants in the RTSQ-available group; 5 male and 10 female participants in the RTSQ-missing group. Fisher's exact test p = .284; OR = 1.99, 95% CI [0.59, 7.75]. DASS-21, ATQ, TFI, and AnTI metacognition scores were jointly missing in the RTSQ-missing group; therefore, comparisons could not be calculated for these variables. Review of source documents identified no skipped page, form-version difference, or data-transfer error.

Panel B. *Inverse-probability-weighted sensitivity analysis for missing RTSQ data*

Model	B	SE	df	95% CI	p
Unweighted complete case	1.638	0.428	168.2	[0.799, 2.476]	< .001
IPW weighted; 1st-99th percentile trimming	1.649	0.427	168.1	[0.813, 2.486]	< .001

Note. B denotes the difference in t0-to-t30 Dangerousness change per 1-SD increase in baseline RTSQ score. Stabilized weights were estimated from observed age, sex, and baseline BTMI subscale scores and trimmed at the 1st and 99th percentiles. The median weight was 0.993, the trimmed range was 0.942-1.113, and the effective sample size was 169.73. This analysis evaluates sensitivity to selection conditional on observed variables; it does not rule out selection related to unobserved characteristics. BTMI = Beliefs Toward Mental Illness Scale.

Supplementary Table S4

Mixed-model diagnostics and influence analyses

Panel A. *Technical diagnostics and ML-REML comparison*

**Check**	**Result**
Number of participants	170
Number of repeated observations	339
Singular fit in REML model	FALSE
Singular fit in ML model	FALSE
Residual skewness	0.002
Residual excess kurtosis	0.779
Shapiro–Wilk p	.107
|Residual|–fitted Spearman ρ	-0.038
|Residual|–fitted Spearman p	.480
Participants flagged in leave-one-out analysis	10

Note. Models differing in fixed effects were compared using ML: adding the time-by-RTSQ interaction improved fit, $\chi^2(1) = 14.30$, $p < .001$. The final coefficient was reported from the REML model. The ML and REML estimates were both B = 1.638.

Panel B. *Summary of participant-level leave-one-out influence diagnostics*

**Check**	**Result**
Full-model time × RTSQ coefficient	1.638
Participants evaluated	170
Flagged participants	10
Minimum coefficient after deleting one participant	1.545
Maximum coefficient after deleting one participant	1.790
Maximum absolute relative change	9.3%
ΔR^2_marginal attributable to time × RTSQ	0.017

Check	Result
f^2 for the time × RTSQ interaction	0.018
Flagged versus other participants: RTSQ	p = .945; q = .989
Flagged versus other participants: baseline Dangerousness	p = .015; q = .077
Flagged versus other participants: observed change	p = .040; q = .100

Note. The time × RTSQ coefficient retained its direction after deleting any single participant. The 10 flagged participants did not have extreme RTSQ scores.

Panel C. *Profile of participants flagged in leave-one-out analysis*

Variable	Not flagged, M (SD)	Flagged, M (SD)	Welch t	p	q
RTSQ t0	70.13 (22.89)	69.10 (45.91)	0.07	.945	.989
Dangerousness t0	19.54 (5.98)	26.00 (6.83)	-2.93	.015	.077
Dangerousness t30	17.29 (5.96)	17.33 (8.59)	-0.01	.989	.989
Dangerousness change: t30 - t0	-2.25 (5.07)	-10.67 (10.31)	2.44	.040	.100
Age	24.27 (1.25)	24.20 (0.79)	0.26	.802	.989

Note. Ten participants were flagged in the leave-one-out analysis. Although nominal differences were observed for baseline Dangerousness and observed change, these differences were not significant after Benjamini-Hochberg correction. Sex distribution also did not differ (Fisher's exact test p = .203).

Supplementary Table S5

HC3-robust ANCOVA sensitivity model

**Predictor**	**B**	**HC3 SE**	**z**	**p**	**95% CI**
Intercept	17.427	0.646	26.98	< .001	[16.161, 18.693]
Baseline Dangerousness	3.441	0.411	8.37	< .001	[2.636, 4.247]
DASS-21	-0.785	0.587	-1.34	.181	[-1.936, 0.365]
ATQ	0.588	0.617	0.95	.341	[-0.622, 1.798]
TFI	0.261	0.528	0.50	.620	[-0.773, 1.295]
RTSQ	1.601	0.531	3.01	.003	[0.560, 2.642]
AnTI metacognition	-0.055	0.741	-0.07	.941	[-1.508, 1.398]
Age	0.089	0.379	0.23	.815	[-0.654, 0.831]
Female sex	-0.324	0.876	-0.37	.711	[-2.041, 1.393]

Note. The outcome is follow-up Dangerousness. Continuous predictors are z-standardized. Male participants are the reference category for sex. The RTSQ coefficient remained significant in the extended model.

Supplementary Table S6

Latent measurement diagnostics and i13 robustness analyses

Panel A. *Ordinal longitudinal measurement-invariance models for Dangerousness*

Model	χ^2	df	CFI	TLI	RMSEA	RMSEA 95% CI	SRMR	$\Delta\chi^2$	Δdf	p	ΔCFI	ΔRMSEA
Configural	121.35	89	0.989	0.985	0.045	[0.021, 0.063]	0.051	—	—	—	—	—
Threshold invariance	145.93	112	0.989	0.988	0.041	[0.018, 0.058]	0.051	23.12	23	.454	-0.001	-0.004
Threshold + loading invariance	155.72	119	0.988	0.988	0.041	[0.020, 0.058]	0.055	11.12	7	.133	-0.001	0.000
Full intercept invariance	186.68	126	0.980	0.981	0.051	[0.035, 0.066]	0.056	31.84	7	< .001	-0.008	0.010
Partial intercept invariance: i01 and i13 freed	168.56	124	0.985	0.986	0.044	[0.025, 0.060]	0.056	13.45	5	.020	-0.003	0.003

Note. Sequential comparisons are based on WLSV scaled difference tests. Full intercept invariance was not supported. The intercepts of i01 and i13 were freed after considering the repeated pattern of local misfit and the adequate fit of the partial-invariance solution.

Panel B. *Residual covariances retained in the latent measurement model*

Indicator 1	Indicator 2	Type	Standardized residual covariance	p
bmi_t0_i01	bmi_t30_i01	Cross-time residual for the same item	0.505	< .001
bmi_t0_i02	bmi_t30_i02	Cross-time residual for the same item	0.374	< .001
bmi_t0_i03	bmi_t30_i03	Cross-time residual for the same item	0.272	.040
bmi_t0_i04	bmi_t30_i04	Cross-time residual for the same item	0.396	< .001
bmi_t0_i05	bmi_t30_i05	Cross-time residual for the same item	0.355	< .001
bmi_t0_i06	bmi_t30_i06	Cross-time residual for the same item	0.391	< .001
bmi_t0_i07	bmi_t30_i07	Cross-time residual for the same item	0.388	< .001
bmi_t0_i13	bmi_t30_i13	Cross-time residual for the same item	0.413	< .001
bmi_t0_i01	bmi_t0_i06	Within-time local dependence	0.556	< .001
bmi_t30_i01	bmi_t30_i06	Within-time local dependence	0.697	< .001
bmi_t0_i02	bmi_t0_i07	Within-time local dependence	0.391	< .001
bmi_t30_i02	bmi_t30_i07	Within-time local dependence	0.500	< .001
bmi_t0_i01	bmi_t30_i06	Cross-time local dependence	0.389	< .001
bmi_t0_i06	bmi_t30_i01	Cross-time local dependence	0.434	< .001

Note. Cross-time residual covariances for the same items were included as a natural feature of the repeated-measures design. Additional local-dependence pairs were retained in the secondary latent route because they showed a repeated pattern during staged local-fit evaluation and were substantively defensible.

Panel C. *Category frequencies for item i13 and rationale for symmetric collapsing*

Time	Category	Frequency	%	Valid n	Missing n
t0	0	50	27.0	185	0
t0	1	57	30.8	185	0
t0	2	40	21.6	185	0
t0	3	32	17.3	185	0
t0	4	5	2.7	185	0
t0	5	1	0.5	185	0
t30	0	55	29.9	184	1
t30	1	56	30.4	184	1
t30	2	42	22.8	184	1
t30	3	25	13.6	184	1
t30	4	6	3.3	184	1
t30	5	0	0.0	184	1

Note. Because no observations were present in category 5 at follow-up and WLSMV estimation is sensitive to sparse or empty category cells, category 5 was collapsed into category 4 in the latent-analysis copy only. The same rule was applied symmetrically at t0 to preserve comparability. The original observed-score analysis was not altered.

Panel D. *Robustness analyses for the category handling of item i13*

**Analytic route**	**Model**	**Effect**	**Estimate**	**SE**	**95% CI**	**p**
Observed score	Original scoring	Time × RTSQ, B	1.638	0.428	[0.799, 2.476]	< .001
Observed score	i13: 5 -> 4 harmonization	Time × RTSQ, B	1.641	0.427	[0.803, 2.478]	< .001
Latent change	Original latent model	RTSQ -> latent change, beta	.337	.089	[.164, .511]	< .001
Latent change	Latent model without i13	RTSQ -> latent change, beta	.345	.093	[.164, .527]	< .001

Note. Applying the symmetric collapsing rule to the observed-score route solely as a sensitivity analysis and excluding i13 from the latent model did not alter the primary RTSQ inference. Fit of the latent model without i13 was adequate: CFI = .983, TLI = .983, RMSEA = .048, SRMR = .060.

Supplementary Table S7

Conditional latent change-score model

Index or parameter	Estimate	SE	z	p	95% CI
Sample size	169	—	—	—	—
Scaled χ^2	191.45	—	—	—	—
df	138	—	—	—	—
CFI	0.981	—	—	—	—
TLI	0.981	—	—	—	—
RMSEA	0.048	—	—	—	[0.030, 0.064]
SRMR	0.061	—	—	—	—
Mean latent change	-0.460	0.082	-5.60	< .001	[-0.621, -0.299]
Variance of latent change	0.645	0.109	5.92	< .001	[0.431, 0.858]
Correlation between baseline level and latent change	-0.409	0.089	-4.58	< .001	[-0.585, -0.234]
RTSQ → latent Dangerousness change (β)	0.337	0.089	3.81	< .001	[0.164, 0.511]
Explained variance in latent change (R^2)	0.114	—	—	—	—

Note. Adding the RTSQ path improved fit relative to the unconditional model, $\Delta\chi^2(1) = 12.95$, $p < .001$. The positive RTSQ coefficient indicates that the reduction in Dangerousness was more limited at higher baseline RTSQ levels.

Supplementary Table S8

Overall Benjamini-Hochberg sensitivity analysis

**Analysis**	**p**	**Within-family q**	**Overall-sensitivity q**
Dangerousness paired change	< .001	< .001	< .001
Helplessness-interpersonal paired change	.014	.019	.055
Shame paired change	.820	.820	.820
BTMI total paired change	< .001	< .001	< .001
Dangerousness: time × RTSQ	.002	.019	.011
Dangerousness: Block 1 -> Block 2	.015	.037	.055

Note. The overall q column is a more conservative sensitivity adjustment that combines the prespecified analysis families. The time × RTSQ interaction for Dangerousness remained supported under this approach (overall q = .011).

Supplementary Table S9

Incremental effect size and power sensitivity analyses

Panel A. *Incremental mixed-model effect size of the time × RTSQ interaction*

Model	Marginal R^2	Conditional R^2	f^2
Reduced model without the interaction	.048	.586	—
Full model with time × RTSQ	.065	.614	—
Increment attributable to time × RTSQ	.017	—	.018

Note. Marginal R^2 represents variance explained by the fixed effects, whereas conditional R^2 represents variance explained jointly by fixed and random effects. The incremental effect size of the time × RTSQ interaction was small.

Panel B. *Power and sensitivity analysis based on realized sample sizes*

Analysis	Realized sample	α	Target power	Minimum detectable effect	Method
Primary mixed-effects model: time × baseline RTSQ	170 participants; 339 repeated observations	.05	.80	Interaction B ≈ 1.20	simr simulation-based sensitivity analysis; ML likelihood-ratio test; 1,000 simulations per final candidate
Paired pre- to post-clerkship Dangerousness comparison	184 paired observations	.05	.80	Cohen's d_z = .208	Analytical sensitivity analysis with pwr.t.test
Baseline-Dangerousness-adjusted ANCOVA: incremental RTSQ contribution	167 participants	.05	.80	Cohen's f^2 = .048	Analytical sensitivity analysis with pwr.f2.test

Note. Because the study was based on a completed longitudinal dataset, these values are sensitivity-analysis results rather than post-hoc observed-power estimates. The mixed-model value is the smallest coefficient in the final grid for which estimated power reached or exceeded .80. In the final grid, estimated power was .744 for B = 1.15 and .817 for B = 1.20; the minimum detectable interaction should therefore be interpreted as approximate. The observed time × RTSQ coefficient was B = 1.638.

Supplementary figures

Supplementary Figure S1. Expected change in Dangerousness by baseline RTSQ level in the age- and sex-adjusted model. Points and error bars represent adjusted simple-slope estimates and 95% confidence intervals. The confidence interval crosses zero at a high RTSQ level.

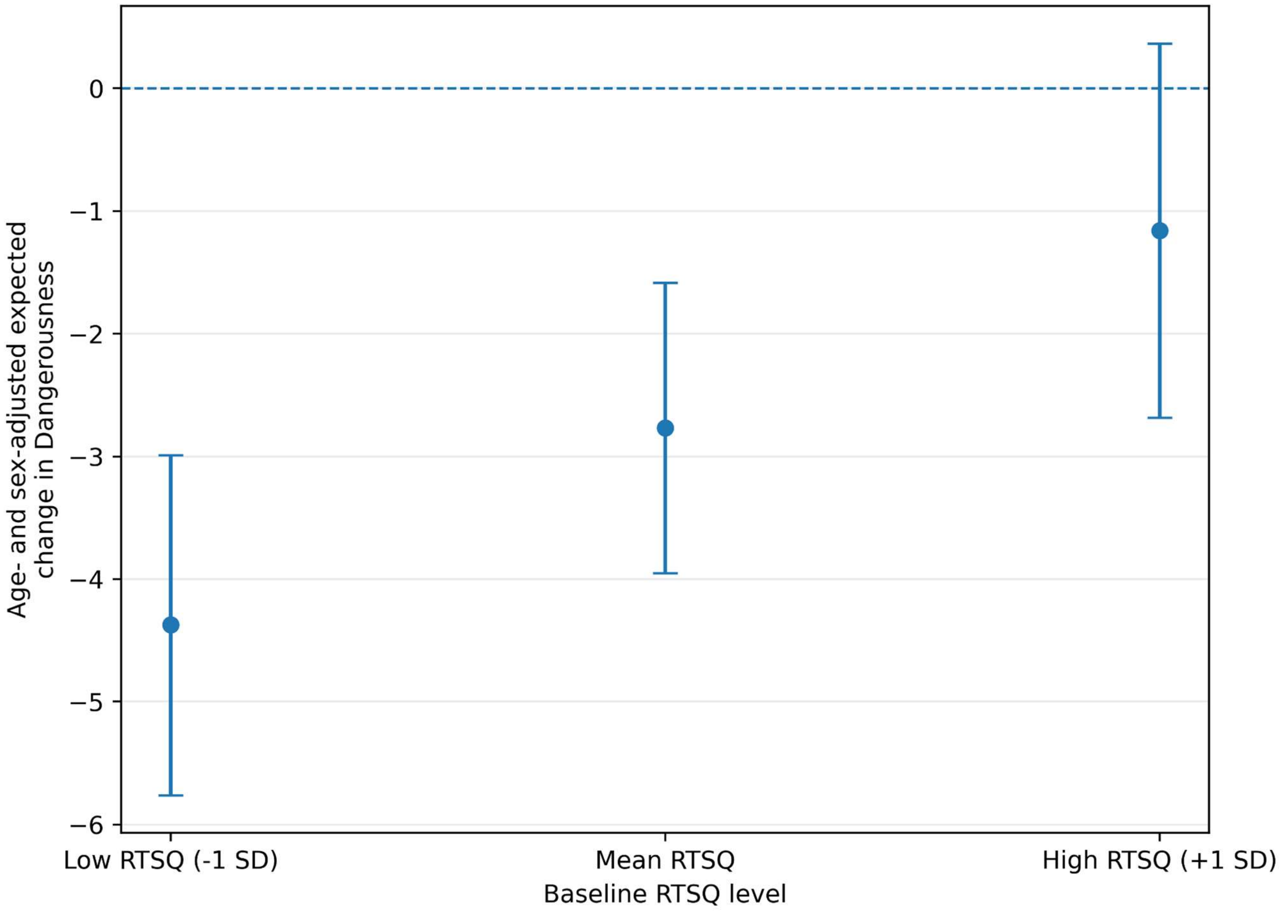

Supplementary Figure S2. Residuals versus fitted values for the final mixed-effects model.

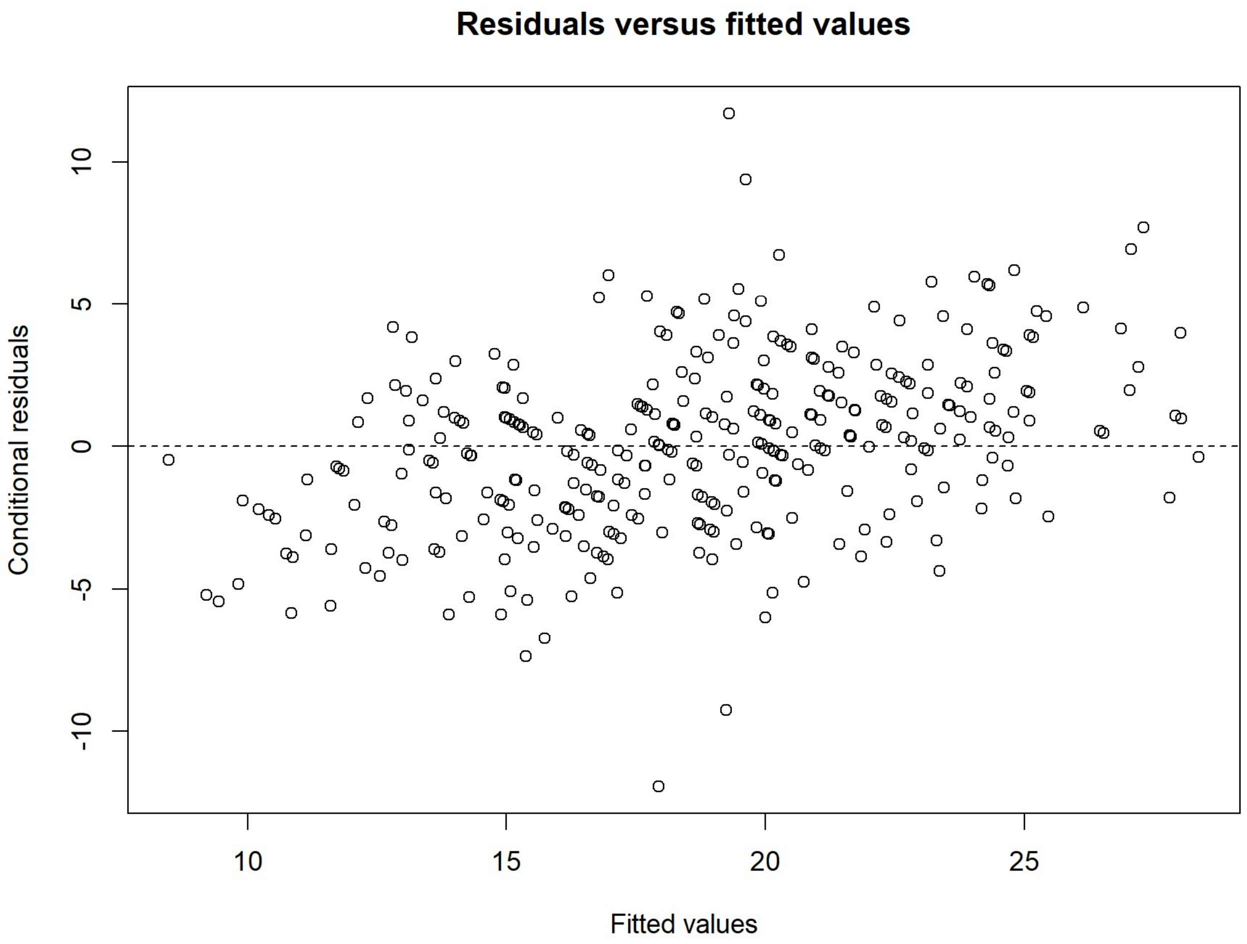

Supplementary Figure S3. Normal Q-Q plot of residuals from the final mixed-effects model.

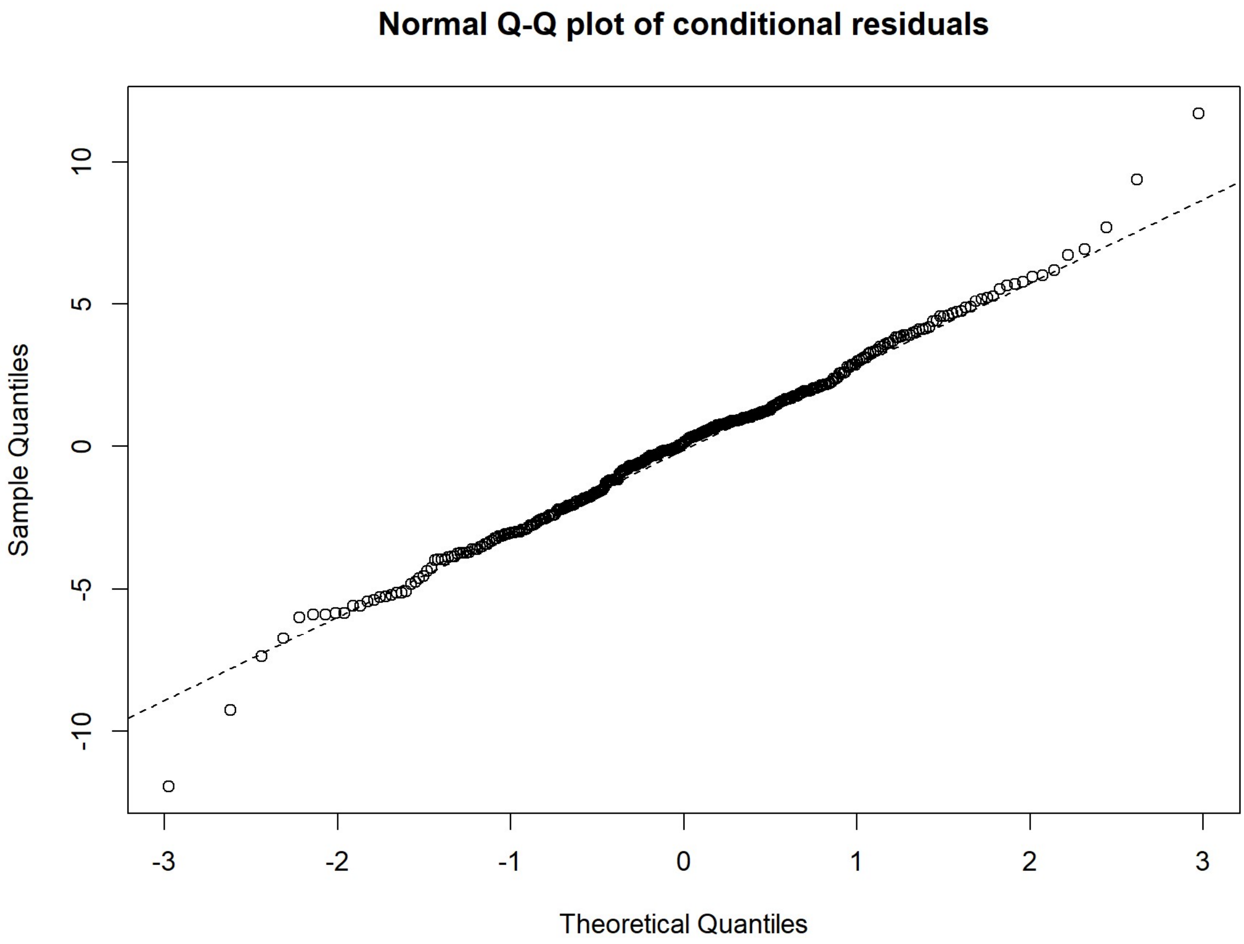

Supplementary Figure S4. Simulation-based sensitivity analysis for the primary mixed-effects model. Points represent estimated statistical power for candidate time × baseline RTSQ interaction coefficients; error bars show 95% binomial confidence intervals. The horizontal dashed line indicates the .80 target-power threshold, and the vertical dotted line indicates the approximate minimum detectable coefficient (B ≈ 1.20). These are sensitivity-analysis results, not post-hoc observed-power estimates.

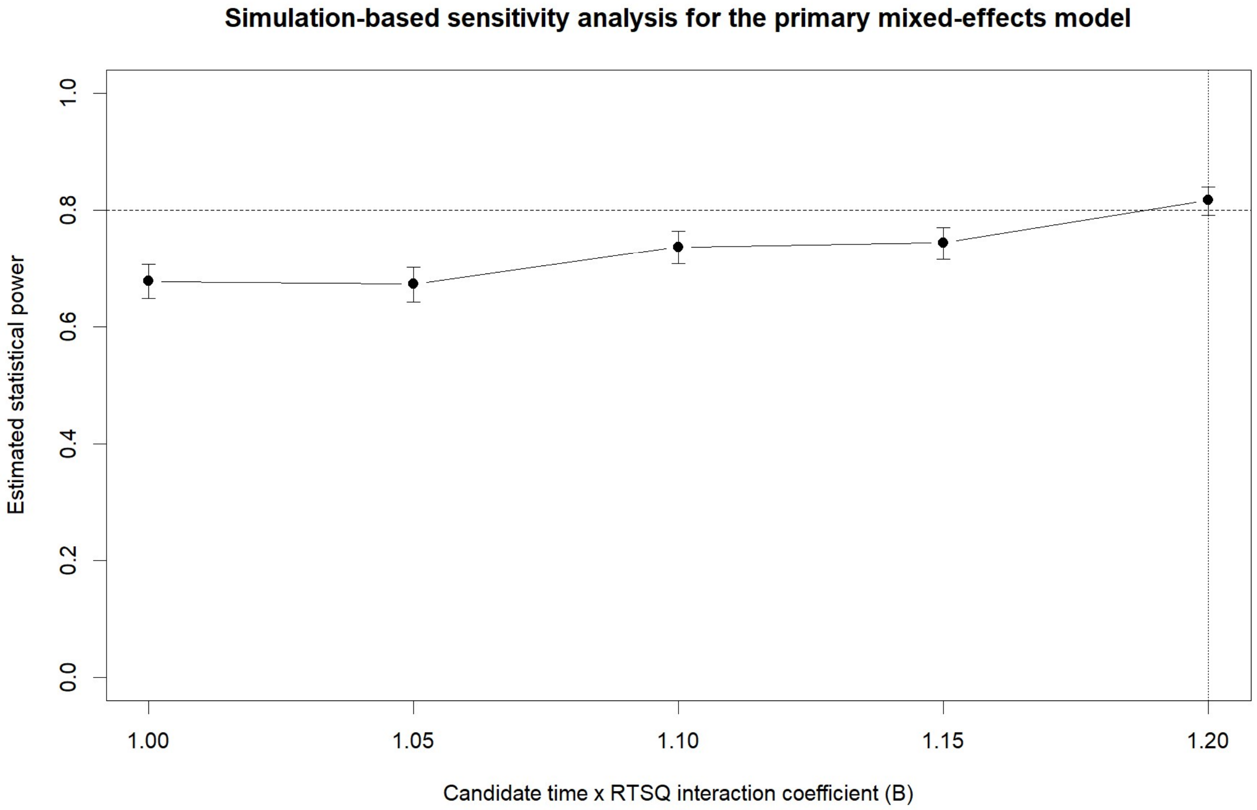

Supplement: Supplementary file 1 [file behavsci-16-01383-s001.zip › behavsci-4422239-supplementary.pdf]
